# Tumor microenvironment and epithelial-mesenchymal transition in bladder cancer: Cytokines in the game?

**DOI:** 10.3389/fmolb.2022.1070383

**Published:** 2023-01-09

**Authors:** Cláudia Martins-Lima, Ugo Chianese, Rosaria Benedetti, Lucia Altucci, Carmen Jerónimo, Margareta P. Correia

**Affiliations:** ^1^ Cancer Biology and Epigenetics Group, Research Center of IPO Porto (CI-IPOP)/RISE@CI-IPOP (Health Research Network), Portuguese Oncology Institute of Porto (IPO Porto) and Porto Comprehensive Cancer Center (Porto.CCC) Raquel Seruca, Porto, Portugal; ^2^ Department of Precision Medicine, University of Campania “Luigi Vanvitelli”, Naples, Italy; ^3^ BIOGEM, Molecular Biology and Genetics Research Institute, Avellino, Italy; ^4^ IEOS, Institute of Endocrinology and Oncology, Naples, Italy; ^5^ Department of Pathology and Molecular Immunology at School of Medicine and Biomedical Sciences, University of Porto (ICBAS-UP), Porto, Portugal

**Keywords:** tumor microenvironment (TME), bladder cancer, cytokines/chemokines, immune cells, epithelial-mesenchymal transition (EMT)

## Abstract

Bladder cancer (BlCa) is a highly immunogenic cancer. Bacillus Calmette-Guérin (BCG) is the standard treatment for non-muscle invasive bladder cancer (NMIBC) patients and, recently, second-line immunotherapies have arisen to treat metastatic BlCa patients. Understanding the interactions between tumor cells, immune cells and soluble factors in bladder tumor microenvironment (TME) is crucial. Cytokines and chemokines released in the TME have a dual role, since they can exhibit both a pro-inflammatory and anti-inflammatory potential, driving infiltration and inflammation, and also promoting evasion of immune system and pro-tumoral effects. In BlCa disease, 70–80% are non-muscle invasive bladder cancer, while 20–30% are muscle-invasive bladder cancer (MIBC) at the time of diagnosis. However, during the follow up, about half of treated NMIBC patients recur once or more, with 5–25% progressing to muscle-invasive bladder cancer, which represents a significant concern to the clinic. Epithelial-mesenchymal transition (EMT) is one biological process associated with tumor progression. Specific cytokines present in bladder TME have been related with signaling pathways activation and EMT-related molecules regulation. In this review, we summarized the immune landscape in BlCa TME, along with the most relevant cytokines and their putative role in driving EMT processes, tumor progression, invasion, migration and metastasis formation.

## Introduction

Urothelial cell carcinoma is the most frequent type of bladder cancer (BlCa), corresponding to approximately 90% of the total cases (Cao et al., 2019). 70–80% of the cases are non-muscle invasive bladder cancer (NMIBC), while the remaining 20–30% are muscle-invasive bladder cancer (MIBC) at the time of diagnosis (Yun and Kim, 2013; Chandrasekar et al., 2018). After receiving surgical treatment, almost half of NMIBC patients experience recurrences once or more, with 5–25% of these patients eventually developing to MIBC, the most severe form of the disease (Kamat et al., 2017). Also, a fraction of patients can show metastases at the time of diagnosis, or develop metastatic disease during follow-up, mainly to the bone (Stellato et al., 2021), distant lymph nodes, lung (Dong et al., 2017) and liver (Wang et al., 2020).

BlCa has the highest cumulative treatment cost, compared to other types of cancers (Bryan, 2015). The standard treatment for NMIBCs, except for carcinoma *in situ* (CIS), is transurethral resection of bladder tumor (TURBT). After TURBT, intravesical immunotherapy *Bacillus* Calmette-Guérin (BCG) is usually applied in order to reduce the risk of recurrence and progression (Kamat et al., 2017; Chandrasekar et al., 2018). BCG has a dual role, since it promotes the activation of the immune system and can directly kill tumor cells (Han et al., 2020). Although the mechanisms of BCG-induced immunotherapy are still incompletely understood (Song et al., 2019), it is known that the immune system is triggered when pathogen-associated molecule patterns (PAMPs), located at the bacterium cell wall, are recognized by pattern recognition receptors (PRRs) expressed by antigen-presenting cells (APCs) and bladder tumor cells. This binding promotes MyD88 signaling pathway stimulation, resulting in nuclear factor kappa-B (NF-kB) activation that promotes cytokine transcription (Han et al., 2020). Additionally, BCG-activated skin dendritic cells (DCs) migrate to the draining lymph nodes to activate adaptive CD4^+^ and CD8^+^ T cells, and activation of B cells leads to the production of antibodies and memory cells in response to the presence of BCG antigens (Covián et al., 2019).

When tumors progress or are diagnosed as localized MIBC, the recommended treatment is cisplatin-based neoadjuvant chemotherapy (NAC) followed by radical cystectomy (Yafi and Kassouf, 2009; Chandrasekar et al., 2018). Moreover, cisplatin-based chemotherapy is the suggested treatment for individuals who have metastases at the time of diagnosis or develop later on (Chandrasekar et al., 2018). However, most of the times, patients do not respond (Galsky et al., 2012; Minoli et al., 2020) or present several comorbidities impeding the usage of neoadjuvant or adjuvant chemotherapy (Inman et al., 2017). This, alongside with the fact that BlCa is considered as an immunogenic cancer, due to its high tumor mutation burden (TMB) and neoantigens (Hu et al., 2021), led to the Food and Drug Administration (FDA) approving several forms of immunotherapy as second-line treatments for metastatic BlCa patients who had not responded to cisplatin-based chemotherapy (Wołącewicz et al., 2020; Du et al., 2021a). Immune checkpoint blockade (ICB) therapies against PD-L1 (such as atezolizumab, durvalumab and avelumab) or against PD-1 (nivolumab and pembrolizumab) are increasingly promising targets in BlCa (Song et al., 2019; Wołącewicz et al., 2020).

## Tumor microenvironment (TME) in BlCa

Bladder tumor microenvironment (TME) has a crucial role in immunotherapy responses (Du et al., 2021a). TME comprise non-cellular components, such as extracellular matrix (ECM) and soluble biological factors or mediators, as cytokines/chemokines, and cellular components, including tumor cells, endothelial cells, stromal cells, and tumor-infiltrating immune cells (TIICs) (Du et al., 2021a; Liu et al., 2021). According to the ESTIMATE algorithm (Yoshihara et al., 2013), patients with high immune score had better prognosis, while patients with high stromal score were associated with shorter survival (Liu et al., 2021). The development of new immunotherapeutic strategies or an improvement in their effectiveness may be aided by a greater comprehension of the bladder TME (Nair et al., 2020).

### TME immune cells in BlCa

Macrophages are one the most abundant immune cells in the TME, including in BlCa (Miyake et al., 2016; Du et al., 2021b). Tumor-associated macrophages (TAMs) secrete several soluble molecules, such as cytokines and chemokines, that directly influence tumor growth, metastasis, and drug resistance (Hanada et al., 2000; Pan et al., 2020). In BlCa, higher amounts of CD68^+^ (pan-macrophage marker) cells, were associated with higher grade and advanced tumors (Huang et al., 2020; Harras and Abo Safia, 2021). Specifically, TAMs (CD68^+^) number was significantly higher in MIBCs comparing with NMIBCs (Hanada et al., 2000; Viveiros et al., 2022) and higher amounts of CD68^+^ cells were significantly associated with poorer disease specific survival (DSS) in bladder peritumoral regions and with worse overall survival (OS) and DSS in bladder intratumoral regions (Viveiros et al., 2022). Co-cultures between macrophages and BlCa cell lines showed an increase in colony formation, cell migration and cell invasion (Huang et al., 2020). TME influence macrophage polarization and, consequently, macrophage function (Miyake et al., 2016). Macrophages can be classified in anti-tumor/proinflammatory (M1) and pro-tumor/anti-inflammatory (M2) (Miyake et al., 2016). M2 macrophages (CD163^+^) are associated with tumorigenesis, tumor growth, angiogenesis, inhibition of immunosurveillance and ECM degradation (Miyake et al., 2016; Du et al., 2021b; Harras and Abo Safia, 2021). TAMs usually display a bias towards an M2-like phenotype (Takeuchi et al., 2016), as observed in BlCa (Viveiros et al., 2022). Indeed, higher ratio of CD163^+^/CD68^+^ macrophages was correlated with advanced BlCa stage and grade (Takeuchi et al., 2016) and higher amounts of CD163+ were significantly associated with worse DSS and OS (Viveiros et al., 2022).

Fibroblasts are one of the most abundant and active cells in the stroma, performing tissue repair functions (Miyake et al., 2016). Cancer-associated fibroblasts (CAFs) contribute to tumor growth, angiogenesis and treatment resistance by secreting specific cytokines (Miyake et al., 2016). Additionally, CAFs secrete several factors, such as collagen, matrix metalloproteinases (MMPs), chemokines and proteases (Miyake et al., 2016; Du et al., 2021b). Du Y *et al.* demonstrated, *in silico*, that CAFs were abundant in bladder TME. Moreover, the authors showed that higher CAF levels enhanced BlCa progression and were associated with lower OS Du et al. (2021b). Other study demonstrated that co-culture between fibroblasts and BlCa cell lines (UMUC3, T24 and 5637) improved tumor cell invasion (Yeh et al., 2015) and have been associated with cisplatin resistance (Long et al., 2019).

Overall T cells (CD3^+^) were significant increase in MIBC tumors, comparing with high-grade NMIBCs, although no differences were found in bladder peritumoral areas (Viveiros et al., 2022). It was shown that CD3^+^ in tumor infiltrating lymphocytes (TILs) were related with poor outcome in BlCa patients (Russo et al., 2022). However, Viveiros N *et al.* proved that an enrichment of CD3^+^ cells, in the intratumoral area, significantly associated with higher disease-free survival (DFS) (Viveiros et al., 2022) and Sjödahl G *et al.* showed that infiltrating CD3^+^ cells were significantly associated with good prognosis in the MIBC cases (Sjödahl et al., 2014).

*In silico*, cytotoxic CD8^+^ T cells correlated with better patient outcome, being observed a decrease of CD8^+^ levels in higher BlCa stages (Cao et al., 2019; Zhang et al., 2020). In patient tissues, Zhang S *et al.* and Jóźwicki W *et al.* reported that CD8^+^ TILs was found mostly in pTa-pT1, comparing with pT2 tumors Jóźwicki et al. (2016), Zhang et al. (2017). Specifically, in Zhang S *et al.* study, higher CD8^+^ was associated with better OS in non-organ confined disease, but with worse OS in organ-confined disease patients, suggesting that cytotoxic T cells might have anti-tumor activity in non-organ confined disease and a pro-tumor activity in organ-confined disease Zhang et al. (2017). Viveiros N *et al.* observed that MIBC patients presented higher CD8^+^ expression, comparing with NMIBC high-grade, but, specifically, MIBC tumors with high intratumoral CD8 expression demonstrated higher DFS and OS Viveiros et al. (2022). Additionally, it was shown that poor CD8^+^ T cell expression, along with type I IFN signature and IFN-inducible inhibitory factors, characterize a non-T cell inflamed bladder TME (Trujillo et al., 2018), usually correlated with poor prognosis and resistance to immunotherapies (Sweis et al., 2016).

*In silico*, Cao J *et al.* observed that CD4^+^ memory resting cells decreased with higher BlCa stage, while CD4^+^ memory activated T cells increased Cao et al. (2019). Zhang Y *et al.* showed, *in silico*, that activated memory CD4^+^ cells were significantly associated with better outcome, while resting memory CD4^+^ cells were associated with poor outcome in BlCa patients Zhang et al. (2020). In BlCa tissues, CD4^+^ levels were significantly higher in pTa-pT1 patients, comparing with most aggressive tumors (Jóźwicki et al., 2016; Viveiros et al., 2022). However, stratifying the tumoral areas, it was observed that CD4^+^ cells were significantly enriched in high-grade NMIBCs in peritumoral area, while CD4^+^ levels were significantly abundant in MIBCs in intratumoral area (Viveiros et al., 2022).

Regulatory T (Treg) cells are a subpopulation of CD4^+^ T cells, characterized by the expression forkhead box protein P3 (FOXP3) transcription factor (Winerdal et al., 2011; Ariafar et al., 2020). Tregs are known to trigger several immunosuppressive mechanisms, both by contact-dependent manner, or indirectly through the secretion of several cytokines, capable of promoting tumor progression (Ariafar et al., 2020). Ariafar A *et al.*, detected a Treg population (CD4^+^CD25+FOXP3+CD127^low/neg^) in lymph nodes from BlCa patients, representing about 10% of all CD4^+^ T cells Ariafar et al. (2020). In this study, Treg cells were significantly higher in patients with at least one involved node, comparing with negative-node patients, although no impact was observed in the survival time (Ariafar et al., 2020), suggesting that Tregs might play a role in tumor metastasis formation (Ariafar et al., 2020). Viveiros N *et al.* observed that Treg cells were significantly lower in the peritumoral area in more advanced stages (pT3 and pT4), but were significantly higher in the intratumoral areas in pTa-pT1 (Viveiros et al., 2022). Moreover, higher Treg amounts in intratumoral areas of high-grade NMIBCs were associated with poor OS and DSS (Viveiros et al., 2022). Jóźwicki W *et al.* showed that Treg amounts were significantly higher in BlCa patients peripheral blood before the surgery, comparing with after surgery (Jóźwicki et al., 2016).

In BlCa, NK cells have been proved to be important in BCG-treatment (Brandau et al., 2001; Esteso et al., 2021), however less is known regarding the role of NK cells in bladder tumor immune surveillance (Sun et al., 2021a). Krpina K *et al.* demonstrated that NMIBC patients with recurrent disease presented significantly higher levels of stromal NK cells, compared with NMIBC patients without recurrence disease (Krpina et al., 2014). Additionally, NMIBC patients with recurrent pTa tumors, recurrent smaller tumors, and recurrent single tumors, presented significantly higher levels of stromal NK cells, than no reccurent NMIBC patients (Krpina et al., 2014). NK cells can be divided in CD56^dim^ NK cells (CD3^−^CD56^dim^CD16^+^), presenting higher cytolytic activity, and in CD56^bright^ NK cells (CD3^−^CD56^bright^CD16^−^), presenting immunoregulatory function through abundant cytokine production (Lin et al., 2004; Poli et al., 2009; Moretta, 2010). In BlCa patients, it was demonstrated that most NK cells were dim NK cells and the proportion of intratumoral dim NK cells were significantly higher in most advanced stages (Mukherjee et al., 2018). Furthermore, higher amounts of CD56^bright^ NK cells were significantly associated with better OS and cancer-specific survival (CSS) (Mukherjee et al., 2018).

DCs are specialized APCs that comprise a rare immune cell population in tumors and in lymphoid organs (Gallo and Gallucci, 2013; Wculek et al., 2020). DCs are essential in trigging antigen-specific immunity and tolerance, since present antigens to T cells and produce immunomodulatory signals by cytokines and cell-cell contacts (Wculek et al., 2020). DCs can be stratified in plasmacytoid (pDC) and in myeloid (mDC) DCs (Martin-Gayo and Yu, 2019). Although DCs are in very low amounts in peripheral blood, Rossi R *et al.* showed a significant decrease of mDCs and pDCs levels in NMIBC patients peripheral blood before TURBT, comparing with healthy donors (Rossi et al., 2013). Also, the authors showed a significant decrease of mDCs in low-grade NMIBC patients before TURBT, compared with high-grade NMIBC patients, while for pDCs no significant differences were observed (Rossi et al., 2013). Patients who received BCG instillations showed peripheral blood evidence of mDC recovery, especially from the third instillation until the completion of the treatment, but no appreciable alterations were detected for pDCs (Rossi et al., 2013). While urine samples did not present mDCs or pDCs before, from third week of BCG instillations mDCs were detected (Rossi et al., 2013). DC cells previously co-cultured with the pumc-91 BlCa cell line resulted in an impaired induction of T cell proliferation. Additionally, a decrease in the levels of T cell-derived cytokines (IL-2, IL-4, IL-6, IL-10, TNF-α, IFN-γ and IL-17A) was observed, compared to control DCs (Xiu et al., 2016), indicating that BlCa cells might induce DC dysfunction, failing to induce T cell responses (Xiu et al., 2016). In patient tissues, high-grade NMIBC and MIBC patients showed similar mature DCs (CD83^+^) levels in bladder peritumoral area and absent expression in intratumoral area (Viveiros et al., 2022).

B cells are important molecules in the adaptive immune response capable of produce both pro- and anti-inflammatory cytokines (Magatti et al., 2020). *In silico* analysis demonstrated that naive B cells were significantly lower in BlCa tumors than in control samples (Zhang et al., 2020). However, Ou Z *et al.* demonstrated that BlCa tissues had more B cells (CD20^+^), than the adjacent normal tissue samples (Ou et al. 2015). Considering high-grade NMIBC and MIBC patients, B cells were only present in bladder peritumoral areas (Viveiros et al., 2022). B cells were significanlty increased in MIBCs, and higher B cell levels were statistically associated with poor DSS (Viveiros et al., 2022). Moreover, Ou Z *et al.* showed that BlCa cell lines’ migration and invasion significantly increase after co-culture with B cells and *in vivo*, tumor infiltrating B cells could promote BlCa metastasis Ou et al. (2015).

Immune cells are major cytokines/chemokine producers, playing a role in initiating and triggering immune responses and recruitment of other cell populations to the tumor site. Thus, dysregulations in immune populations in the tumor, can then reflect in the cytokine production in the TME. Those alterations will not only impact in the recruitment and shaping of other immune cells, but also in shaping tumor cells. The impact of TME on driving tumor cell mechanisms that lead to evasion will define tumor development.

## Epithelial-mesenchymal transition (EMT) in BlCa

Epithelial-mesenchymal transition (EMT) is a process involved in tumor progression. EMT can be divided in three different types, according to the biological context (Kalluri and Weinberg, 2009). EMT type 1, occurs during embryogenesis, while EMT type 2 relates with inflammation process, wound healing and tissue regeneration (Kalluri and Weinberg, 2009; Yun and Kim, 2013). EMT type 3 is usually associated with tumor progression, particularly in NMIBC to MIBC progression (Kalluri and Weinberg, 2009; Cao et al., 2020). Traditional EMT involves cellular transdifferentiation, which causes changes in desmosomes, adherens junctions, and tight junctions in epithelial cells. A change in the actin cytoskeletal architecture during this phase results in phenotypical changes where front-rear polarity replaces apical-basal polarity. (Koo et al., 2010; Lu and Kang, 2019). Molecularly, it occurs a decrease in epithelial-related genes, such as *CDH1*, *TJP1*, *CLDN1* and specific cytokeratin genes, and an increase in mesenchymal-related genes, such as *VIM*, *CDH2*, *ITGB1* and *ITGB2* (Koo et al., 2010; Lu and Kang, 2019). Additionally, cells exhibiting EMT characteristics can degrade the extracellular matrix by MMPs (Xu et al., 2009; Lu and Kang, 2019). As a result, these cells increase motility, develop resistance to apoptosis, and become isolated, which culminates in cell invasion and migration (Xu et al., 2009; Koo et al., 2010). According to *in silico* analysis, EMT signaling pathways were shown to be significantly activated from NMIBCs to MIBCs (Cao et al., 2020). In this same study, low-risk score patients (based on EMT-related gene signature) showed significantly higher OS and DFS rates than high-risk score, and MIBC samples showed a higher risk-score, comparing with NMIBC patients (Cao et al., 2020). Indeed, in BlCa patient samples, *CDH1* and *TP63* transcript levels were significantly higher in superficial tumors, comparing with MIBCs, while in the most aggressive tumors, *VIM*, *ZEB1*, *ZEB2*, *MMP2* and *MMP9* transcript levels were significantly enhanced (Choi et al., 2012).

It is becoming increasingly evident that cells can undergo rather a partial EMT, exhibiting hybrid epithelial and mesenchymal features (Lu and Kang, 2019). EMT plasticity involves several epigenetic and genetic alterations, resulting in alterations in the expression of epithelial and mesenchymal markers (Sinha et al., 2020). Cells under partial EMT demonstrate several advantages, comparing with cells with complete EMT phenotypes, such as higher survival mechanisms, tumor-initiating and metastatic potential, which might enhance immune-resistance and chemo-tolerance and increase tumor aggressiveness (Jolly et al., 2015). Indeed, it was shown that there is a “cadherin modulation” in advanced BlCa, where the epithelial marker E-cadherin is expressed at lower levels, simultaneously with high levels of mesenchymal-associated P-cadherin and/or R-cadherin (Martins-Lima et al., 2022).

According to the literature, partial EMT is maintained by phenotypic stability factors (PSFs) and several EMT-inducing transcription factors (EMT-TFs) (Bocci et al., 2019; Sinha et al., 2020). The most well-known EMT-TFs are the zinc-finger-binding transcription factors Snail and Slug, the basic helix-loop-helix (bHLH) factor TWIST1, and the zinc-finger E-box-binding homeobox factors ZEB1 and ZEB2 (Kalluri and Weinberg, 2009; Jolly et al., 2015). Usually, these EMT-TFs are responsible for *CDH1* repression and *CDH2* expression (Wendt et al., 2009). There are specific signaling pathways related with EMT induction, such as transforming growth factor β (TGF-β), bone morphogenetic protein (BMP), Notch, Wnt, hepatocyte growth factor (HGF), epidermal growth factor (EGF), fibroblast growth factor (FGF), platelet-derived growth factor (PDGF), sonic hedehog (Shh), and integrin signaling (Xu et al., 2009; Gonzalez and Medici, 2014; Jolly et al., 2015; Lu and Kang, 2019).

## TME cytokines/chemokines in BlCa and impact in EMT modulation

TME has been described to have an important role, not only in EMT induction, but also in the reversion process, mesenchymal-epithelial transition (MET), in distant metastasis (Sinha et al., 2020). Immune cells, besides playing fundamental direct anti-tumoral and pro-tumoral roles, can also display their function through the secretion of cytokines (Zhang and An, 2007; Shelton et al., 2021). Moreover, other types of cells, as endothelial cells, tumor cells, and fibroblasts, are able to produce cytokines (Dunlop and Campbell, 2000; Zhang and An, 2007; Van Linthout et al., 2014). Cytokines are small secreted proteins that participate in cell-cell interaction and communication (Zhang and An, 2007). Cytokine-target cells can be cells that secrete them, in an autocrine action, or the distant cells, in an endocrine action (Zhang and An, 2007). Several cytokines can display both anti-inflammatory and pro-inflammatory potential (Ramesh et al., 2013). Although cytokines participates in tissue damage control and repair (Suarez-Carmona et al., 2017), these soluble molecules can also modulate the TME and, consequently, shape tumor biology (Morizawa et al., 2018), promoting tumor cell survival, proliferation, angiogenesis and immunosuppression (Suarez-Carmona et al., 2017). According to their function and structure, cytokines can be stratified into interferons (IFNs), interleukins (ILs), tumor necrosis factor-alpha (TNFs), transforming growth factors (TGFs), chemotactic cytokines (chemokines), and colony-stimulating factors (CSFs) (Kartikasari et al., 2021).

Chemokines play important roles in inflammatory responses, promoting the recruitment of immune cells responsible for innate and adaptive immune responses (Miyake et al., 2013). There are four chemokine groups, based on two cysteine residue positions, XC, CC, CX3C and CXC (Sokol and Luster, 2015; Kohli et al., 2022). CXC chemokine family can be stratified based on the presence of three amino acid residues (Glu-Leu-Arg; ELR motif), comprising CXCL1, CXCL2, CXCL3, CXCL5, CXCL6, CXCL7, and CXCL8, which are powerful angiogenic molecules and presenting neutrophils chemoattraction abilities (Kawanishi et al., 2008). On the other hand, CXCL4, CXCL9 and CXCL10 are chemokines without ELR motif, displaying chemoattraction capacities for mononuclear cells and can inhibit angiogenesis (Addison et al., 2000; Kawanishi et al., 2008). Chemokines can be cleaved by several molecules, such as, MMPS, cathepsins, thrombin, plasmin and elastase (Hughes and Nibbs, 2018). Chemokines and their receptors can play anti-tumor roles, since these molecules are responsible for the recruitment of immune cells to TME, such as CD8^+^ T cells, T helper cells and NK (Chow and Luster, 2014; Bule et al., 2021; Kohli et al., 2022). However, chemokine ligands and receptors can play pro-tumoral roles, namely by recruiting pro-tumorigenic immune, such as tumor-associated neutrophils (TAN), TAMs and Treg cells (Bule et al., 2021). Thus, cytokines might also be implicated in the tumor initiation, growth, progression and involved in metastasis formation (Chow and Luster, 2014; Burnier et al., 2015; Kohli et al., 2022).

According to the literature, specific cytokines have been described to be responsible for the transcriptional activation of several genes, including EMT-related genes (Sistigu et al., 2017), consequently contributing to promote BlCa progression, invasion, migration, metastasis formation and angiogenesis (Inoue et al., 2000; Mian et al., 2003; Tsui et al., 2013; Goulet et al., 2019; Zou et al., 2019). Herein, we will focus on some of the most relevant cytokines/chemokines described to be involved in BlCa tumorigenesis and progression and their putative roles in driving EMT processes.

### IL-8/CXCL8

IL-8, also known as CXCL8, is an angiogenic factor associated with inflammation and tumorigenesis and it is considered a pro-inflammatory cytokine (Urquidi et al., 2012; Yao et al., 2020). This chemokine has a powerful leukocyte chemoattraction (Koçak et al., 2004; Jovanović et al., 2010), specially neutrophils attraction (Jovanović et al., 2010). Indeed, in inflammatory regions, IL-8 is responsible to attract and activate neutrophils (Bickel, 1993). Additionally, IL-8 promotes the adhesion of monocytes and neutrophils to endothelial cells, facilitating translocation to inflamed tissues (Gonzalez-Aparicio and Alfaro, 2018). IL-8 can be secreted by lymphocytes, neutrophils, macrophages and by several types of tumor cells (Ou et al., 2015). Furthermore, IL-8 plays an important role in promoting angiogenesis, since contributes to the growth and survival of endothelial cells (Tseng-Rogenski and Liebert, 2009). CXC chemokine receptor 1 (CXCR1) and CXC chemokine receptor 2 (CXCR2), also known as interleukin-8 receptor type beta (IL8RB), are IL-8 receptors, usually expressed in neutrophils and granulocytic myeloid-derived suppressor cells (GR-MDSC) (Miyake et al., 2019; Teijeira et al., 2020). When IL-8 binds to CXCR1 and CXCR2 activates serine/threonine kinases, protein tyrosines and Rho-GTPases, stimulating the expression of proteins related with cell proliferation, survival and cell invasion (Escudero-Lourdes et al., 2012).

*In silico* GSE32894 database, lower *IL8* levels were associated with improved DSS (Chen et al., 2022). However, in The Cancer Genome Atlas (TCGA) database, it was demonstrated that higher *IL8* levels were significantly associated with basal subtype (usually associated with advanced stage tumors and metastatic disease), comparing with luminal subtype (predominantly associated with papillary histopathological features) (McConkey and Choi, 2018; Chen et al., 2022) (Table 1).

**TABLE 1:** Cytokines/chemokines levels are deregulated during BlCa progression, growth, invasion, and metastases formation.

	IL-8/CXCL8	CCL2	CXCL1	CXCL12	IL-6	TGF-β1
Receptors	CXCR1; CXCR2/IL8RB (Miyake et al., 2019; Teijeira et al., 2020)	CCR2; CCR4 (Zhang et al., 2010; Gao et al., 2019)	CXCR2 (Kawanishi et al., 2008)	CXCR4; CXCR7 (Shen et al., 2013; Zhang et al., 2018)	IL-6R (Andrews et al., 2002)	TGF-βRI; TGF-βRII (Kim et al., 2001)
						
Major producing cells	Tumor cells; Lymphocytes; Neutrophils; Macrophages (Ou et al., 2015)	Tumor cells; Macrophages; Fibroblasts; Lymphocytes; Vascular Smooth Muscle (Amann et al., 1998)	Macrophages; Mast cells (De Filippo et al., 2013)	Cancer associated fibroblasts (Du et al., 2021c)	T lymphocytes; Macrophages; Tumor cells; Endothelial cells; Epithelial cells; Muscle cells (Andrews et al., 2002; Rossi et al., 2015; Schuettfort et al., 2022)	Regulatory T cells; Cancer-associated fibroblasts; M2 macrophages; MDSC (Ao et al., 2007; Yu et al., 2014; Yeh et al., 2015; Groth et al., 2019; Efiloğlu et al., 2020; Horibe et al., 2021)
Urine	↑ in BlCa patients than controls (Urquidi et al., 2012; Al-biaty, 2015; Kumari et al., 2017); ↑ in MIBC tumors (Al-biaty, 2015); ↑ in undifferentiated tumors (Al-biaty, 2015; Kumari et al., 2017); ↑ in recurrent disease (Al-biaty, 2015; Kumari et al., 2017)	↑ in pT2-pT4 than pT1 (Amann et al., 1998)	↑ in BlCa patients than controls (Kawanishi et al., 2008; Burnier et al., 2015); ↑ in pT1-pT4 than pTa (Kawanishi et al., 2008)	↓ *CXCL12A* in lower grade (Gosalbez et al., 2014); ↑ *CXCL12B* in higher grade (Gosalbez et al., 2014); CXCL12G was not detected (Gosalbez et al., 2014)	↑ in pT3-pT4 than patients with early stages or than non-malignant disease (Chen et al., 2013); ↑ IL-6 in lower grades (Kumari et al., 2017); ↑ IL-6 associated with ↓ OS (Morizawa et al., 2018)	↑ in BlCa patients than controls or chronic cystitis disease (Helmy et al., 2007)
						
						
						
*In vitro*	IL-8 promotes cellular growth and cellular survival in normal urothelial cells (Tseng-Rogenski and Liebert, 2009)	↑ in high-grade BlCa cell lines (Chiu et al., 2012); ↓ in low-grade BlCa cell lines (Chiu et al., 2012)	↑ in most aggressive BlCa cell lines (Kawanishi et al., 2008); ↑ CXCL1 increases invasive abilities of BlCa cell lines (Kawanishi et al., 2008; Miyake et al., 2019); ↑ CXCL1 increases angiogenesis abilities of BlCa cell lines (Miyake et al., 2019)	Regulates BlCa cell invasion abilities (Shen et al., 2013); Regulates BlCa cell migration abilities (Retz et al., 2005)	IL-6 was associated with BlCa cell line invasion (Yeh et al., 2015); IL-6 was associated with BlCa cell line growth/proliferation (Okamoto et al., 1997; Miyake et al., 2019)	TGF-β1 was associated ↑ BlCa cell line proliferation; TGF-β1 was associated ↑ BlCa cell line colony formation; TGF-β1 was associated ↑ BlCa cell line invasion; TGF-β1 was associated ↑ BlCa cell line migration (Bian et al., 2013; Zhang et al., 2016; Zou et al., 2019)
						
						
Patient tissues	↓ in BlCa patients (Reis et al., 2012); ↑ in undifferentiated tumors (Reis et al., 2012); ↑ in pT1-pT2 than pTa (Reis et al., 2012); ↑ in recurrent disease (Reis et al., 2012)	↑ in BlCa patients than normal/adjacent tissues (Wang et al., 2017); ↑ in undifferentiated tumors (Gao et al., 2019); ↑ in higher stage tumors (Gao et al., 2019); ↑ in lymph node metastasis (Gao et al., 2019); In MIBC patients, ↑ CCL2 in tumor cells was associated with ↓ OS, ↓ DSS and ↓RFS (Eckstein et al., 2020)	Normal or benign tissues did not express CXCL1 (Kawanishi et al., 2008; Miyake et al., 2013); ↑ in undifferentiated tumors (Miyake et al., 2013); ↑ in higher stage tumors (Kawanishi et al., 2008; Miyake et al., 2013); ↑ CXCL1 was associated with ↓ OS (Miyake et al., 2013); ↑ CXCL1 was associated with ↓ DSS (Miyake et al., 2013)	↑ in BlCa patients (Yang et al., 2015) vs. ↓ in BlCa patients (Du et al., 2021c); ↑ in undifferentiated tumors (Batsi et al., 2014); ↑ in higher stage tumors (Batsi et al., 2014); ↑ in recurrent disease (Batsi et al., 2014); Normal tissue did not express *CXCL12* (Yang et al., 2015)	↑ IL-6/*IL6* in BlCa patients than normal tissues or cystitis patients (Chen et al., 2013); ↑ in early stages than non-malignant disease (Chen et al., 2013); ↑ was mostly associated in MIBC tissues (Chen et al., 2013); IL-6 is expressed in non-malignant tissues (Chen et al., 2013)	↓ in normal urothelium (Yang et al., 2018; Zou et al., 2019); ↑ in higher stage tumors (Kim et al., 2001; Yang et al., 2018; Stojnev et al., 2019; Zou et al., 2019); ↑ in undifferentiated tumors (Zou et al., 2019; Stojnev et al., 2019); ↑ was correlated with ↑ cancer-specific death (Stojnev et al., 2019) vs.↑ *TGFB1* in lower stage tumors (Miyamoto et al., 1995); ↑ *TGFB1* in well-differentiated tumors (Miyamoto et al., 1995); ↑ TGF-β1 in BlCa tumors than normal tissues (Miyamoto et al., 1995)
*In silico*	↓ *IL8* was associated with ↑ DSS (Chen et al., 2022); ↑*IL8* was associated with basal subtype (Chen et al., 2022)	↓ *CCL2* in BlCa patients than the controls (Li et al., 2021); ↑ *CCL2* associated with better DFS (Li et al., 2021)	↑ *CXCL1* in BlCa tumors than controls (Sun et al., 2021b); ↑ *CXCL1* was associated with ↓ OS (Sun et al., 2021b)	↓ *CXCL12* in BlCa tumors than controls (Sun et al., 2021b; Du et al., 2021c) vs. In tumors, ↑ *CXCL12* was associated with ↑ stage (Sun et al., 2021b; Liu et al., 2021); In tumors, ↑ *CXCL12* was associated with ↑ lymph node (N2 than N0) (Liu et al., 2021); In tumors, ↑ *CXCL12* was associated with ↓ prognosis (Sun et al., 2021b; Liu et al., 2021)	↑ in undifferentiated tumors (Goulet et al., 2019); ↑ in advanced tumors (Goulet et al., 2019)	↑ *TGFB1* in MIBCs, comparing with NMIBCs (Zou et al., 2019); ↑ *TGFB1* was associated with ↑ risk of death (Zou et al., 2019); ↑ *TGFB1* was associated with ↓ DFS (Zou et al., 2019); ↑ *TGFB1* was associated with ↓ OS (Zou et al., 2019)
*In vivo*	IL-8 regulates tumor growth (Mian et al., 2003); IL-8 regulates BlCa tumorigenicity (Inoue et al., 2000); IL-8 regulates metastasis formation (Inoue et al., 2000); IL-8 regulates neovascularization (Inoue et al., 2000)	Not reported	CXCL1 promotes tumor growth (Miyake et al., 2016); CXCL1 promotes bladder tumor cells attachment to the bladder wall (Miyake et al., 2016); CXCL1 influences proliferation (Miyake et al., 2019); CXCL1 influences angiogenesis (Miyake et al., 2019); CXCL1 influences apoptosis (Miyake et al., 2019)	Influences BlCa cell growth (Zhang et al., 2018)	IL-6 was associated with tumor growth/proliferation (Chen et al., 2013); IL-6 was associated with tumor invasion (Chen et al., 2013); IL-6 was associated with angiogenesis (Chen et al., 2013)	TGF-β1 was associated with ↑ tumor size (Zou et al., 2019); TGF-β1 was associated with ↑ tumor weight (Zou et al., 2019)
Serum	IL-8 expression was associated with ↓ CSS (Morizawa et al., 2018); IL-8 expression was associated with ↓ OS (Morizawa et al., 2018)	Not reported	Not reported	Not reported	↑ IL-6 in recurrent patients than non-recurrent patients (Kumari et al., 2017); ↑ IL-6 in poor RFS (Kumari et al., 2017); IL-6 was associated with ↓ CSS (Morizawa et al., 2018); ↓ T2-T4 patients than Ta-T1 patients and controls (Yang et al., 2017)	↑ TGF-β1 related with ↓ risk tumor progression (Efiloğlu et al., 2020); ↓ TGF-β1 in pT4 than superficial and invasive tumors (pT2-pT3) (Eder et al., 1996) vs.
						↑ TGF-β1 related with ↑ tumor grade and aggressiveness (Eder et al., 1997); ↑ TGF-β1 related with superficial tumors (pTa-pT1) than normal samples (Eder et al., 1997)
						
						
Plasma	Not reported	Not reported	Not reported	Not reported	↑ IL-6/IL-6sR median levels in advanced patients (Andrews et al., 2002; Schuettfort et al., 2022); ↑ IL-6/IL-6sR median levels in lymph vascular invasion (Andrews et al., 2002; Schuettfort et al., 2022); ↑ IL-6/IL-6sR median levels in lymph node metastasis (Andrews et al., 2002; Schuettfort et al., 2022); ↑ IL-6/IL-6sR median levels in recurrent disease (Schuettfort et al., 2022); ↑ IL-6/IL-6sR median levels in patients who deceased from BlCa (Schuettfort et al., 2022); ↑ IL-6/IL-6sR median levels associated with ↓ OS, ↓ RFS and ↓CSS (Schuettfort et al., 2022); ↑ IL-6 in BlCa patients than healthy patients (Andrews et al., 2002)	↑ in MIBC patients (Shariat et al., 2001); ↑ in MIBC patients with regional and distant lymph node (Shariat et al., 2001); ↑ related with ↑ risk of disease recurrence (Shariat et al., 2001); ↑ related with ↑ mortality (Shariat et al., 2001)
EMT-related molecules	*IL8* silencing promoted ↓ *MMP9* (Escudero-Lourdes et al., 2012); IL-8 treatment suppresses E-cadherin, while ↑ Vimentin, ↑ Snail, ↑ Slug and ↑ Twist (Zhou et al., 2021); ↑ IL-8 promoted ↑ MMP-1 and ↑ MMP-13 (Ou et al., 2015); IL-8 regulates *MMP9*/MMP-9 and MMP-2 (Inoue et al., 2000; Mian et al., 2003)	↑ CCL2 promoted ↑ MMP-9, ↑ N-cadherin, ↑ Twist, ↑ Snail and ↑ Vimentin (Rao et al., 2016)	Overexpression of CXCL1 in TAMs and CAFs, promoted ↓ E-cadherin and ↑ MMP-2 (Miyake et al., 2016); A significant correlation was established between CXCL1 and MMP-13 (Kawanishi et al., 2008)	Inhibition of CXCR4 promoted ↓ β-catenin, ↓ MMP-2 and ↓ c-Myc and ↑ E-cadherin levels (Zhang et al., 2018); CXCL12/CXCR4 inhibition promoted ↓ E-cadherin and ↑ c-Myc (Zhang et al., 2018); CXCL12/CXCR4 seems to be important in β-catenin regulation (Zhang et al., 2018)	↑ *IL6* promoted ↓ N-cadherin and ↓ Vimentin levels (Tsui et al., 2013); ↓ *IL6* led to ↓ E-cadherin, but ↑ N-cadherin and ↑ Vimentin levels (Tsui et al., 2013) vs. ↓ *IL6* led to ↑ E-cadherin, but ↓ MMP9 (Chen et al., 2013)	↑ TGF-β1 levels promoted ↓ E-cadherin (Chen et al., 2014, Zou et al., 2019), ↓ *miR-200b* (Chen et al., 2014), ↑ N-cadherin (Chen et al., 2014), ↑ Vimentin (Chen et al., 2014, Zou et al., 2019), ↑ MMP-2 (Zou et al., 2019), ↑ MMP-9 (Zou et al., 2019), ↑ Snail (Zou et al., 2019), and ↑ MMP-16 (Chen et al., 2014)
						
						
						
EMT-related signaling pathways	Overexpression of IL-8 promoted ERK, AKT and STAT3 pathways activation (Zhou et al., 2021); IL-8 regulates the expression of MMPs by NF-kB (Mian et al., 2003)	CCL2-CCR2 interaction may facilitate migration by phosphorylating paxillin y118 through a protein kinase C (PKC)-dependent mechanism (Chiu et al., 2012)	Not reported	CXCL12/CXCR4 promotes STAT3 phosphorylation, resulting in BlCa invasion (Shen et al., 2013)	EMT-player alterations, induced by IL-6, might be regulated by STAT3 signaling pathway activation (Chen et al., 2013);	TGF-β1 promoted an increase in p-Smad2/3 levels (Geng et al., 2014)
					E-cadherin expression might be inhibited by IL6-STAT3 signaling pathways (Chen et al., 2020);	
					IL-6-induced STAT3 activation, being able to target *TWIST* promoter (Yao et al., 2020)	

IL-8 urinary protein concentration was found to be significantly higher in bladder tumor patients, comparing with healthy controls (Urquidi et al., 2012; Al-biaty, 2015; Kumari et al., 2017). Furthermore, a significant IL-8 increase was assessed in higher grade and in MIBC tumors, where recurrent disease showed higher IL-8 protein levels, compared with healthy control or newly diagnosed patients (Al-biaty, 2015; Kumari et al., 2017) (Table 1).

Reis ST *et al.* demonstrated that the majority of bladder tumors tissues underexpressed IL-8, comparing with controls (Reis et al., 2012). However, a significant association was established between high-grade tumors and higher *IL8* levels (Reis et al., 2012). Moreover, pT1 and pT2 showed higher *IL8* levels expression than pTa tumors, and recurrent disease patients demonstrated significant higher *IL8* levels, compared to patients that not recurred (Reis et al., 2012) (Table 1).

It was also demonstrated *in vitro* that IL-8 is actually expressed by normal urothelial cells and promotes not only cellular growth, through AKT pathway, but also cellular survival in normal urothelial cells (Tseng-Rogenski and Liebert, 2009). Additionally, *IL8*/IL-8 levels were significantly higher in BlCa cell lines (J82 and TCCSUP) after co-culture with macrophages (Huang et al., 2020). Furthermore, studies *in vitro* suggest a relationship between IL-8 and BCG treatment, since this treatment promotes Ca^2+^ signaling stimulation and NF-kB activation, being responsible for an increase of IL-8 secretion (Ibarra et al., 2019) (Table 1).

According to the literature, in serum samples, IL-8 expression was significantly associated with poor CSS and shorter OS (Morizawa et al., 2018) (Table 1).

*In vivo* studies demonstrated that IL-8 is able to regulate BlCa tumorigenicity and metastasis formation, and higher IL-8 expression was correlated with higher tumor-induced neovascularization (Inoue et al., 2000). Furthermore, when nude mice implanted with 253J B-V and UMUC3 cell lines in the bladder cell wall were treated with ABX-IL8, an inhibitor of IL-8, it was observed a significant suppression in tumor growth (Mian et al., 2003) (Table 1).

Since IL-8 is upregulated in MIBC tumors (Al-biaty, 2015), and seems to promote tumor growth (Mian et al., 2003) and metastasis formation (Inoue et al., 2000), it suggests that it might play a crucial role in driving EMT. Until now, there are some studies focusing on how deregulation of IL-8 in BlCa might promote alterations in EMT-related molecules and which signaling pathways might be involved in BlCa. It is established that arsenic (As) exposure is a risk factor of BlCa (Escudero-Lourdes et al., 2012). UROtsa, an urothelial cell line, exposed to the arsenic metabolite monomethylarsonous [MMA (III)] undergo malignant transformation. MMA (III) exposure induced *IL8*/IL-8 overexpression, followed by an increase of *CCND1*, *BCL2* and *MMP9* (Escudero-Lourdes et al., 2012). *In vivo*, *IL8* silencing induced a significant decrease of cell proliferation and of tumor formation, while, *in vitro*, was observed a downregulation of *CCND1*, *BCL2* and *MMP9* (Escudero-Lourdes et al., 2012). Furthermore, SVHUC1, a non-malignant BlCa cell line, demonstrated HER2 overexpression and an *IL8*/IL-8 activation upon exposure to As (Zhou et al., 2021). Consequently, IL-8 promoted extracellular signal-regulated kinase (ERK), AKT, and signal transducer and activator of transcription (STAT) 3 signaling activation, resulting in an evident influence in EMT, since the E-cadherin decreased, while Vimentin, Snail, Slug and Twist increased (Zhou et al., 2021). It was shown that a tight junction protein family member, occludin, regulated angiogenesis by controlling IL-8/STAT3 signaling pathway by STAT4 activation (Yang et al., 2022). Retz MM *et al.* showed that co-culture of B cells with the BlCa cell lines, TCCSUP, T24 and J82, increased bladder cell invasion and migration (Ou et al., 2015). The authors suggested that infiltrating B cells can promote IL-8 increase and, consequently, an increase of androgen receptor (AR), leading to MMP-1 and MMP-13 increase (Ou et al., 2015). Corroborating these findings, *in vivo* experiments showed that infiltrating B cells could increase BlCa cell invasion *via* increasing AR signal (Ou et al., 2015). Furthermore, it was demonstrated that IL-8 regulates *MMP9* expression in 253J-P and 253J-BV cells lines (Inoue et al., 2000). Indeed, Mian BM *el al.* showed, *in vitro*, that IL-8 neutralization resulted in a decrease of MMP-2 and MMP-9 expression, in part, through NF-kB, and, consequently, promoted cell invasion decrease (Mian et al., 2003) (Table 1).

### CCL2

Monocyte chemoattractant protein -1/chemokine (C-C motif) ligand 2 (MCP-1/CCL2) plays a crucial role in immune responses, regulating infiltration and migration of several immune cells (Xu et al., 2021). CCL2 is a potent chemoattractant for monocytes/macrophages (Li and Tai, 2013) and can activate dendritic cells, memory T cells and basophils (Chiu et al., 2012; Xu et al., 2021). CCL2 is secreted by activated macrophages, fibroblasts, vascular smooth muscle, lymphocytes, and tumor cells (Amann et al., 1998). Usually binds to C-C chemokine receptor type 2 (CCR2), but it also binds to CCR4 (Zhang et al., 2010; Gao et al., 2019). CCL2 expression can be activated by several growth factors and cytokines, such as platelet-derived growth factor (PDGF), TNF-α, IL-1β and IFN-γ (Li and Tai, 2013). Overall, according to the literature, CCL2 in the TME seems to mainly contributes for tumor progression and metastasis formation (Jin et al., 2021).

*In silico* data analysis showed that *CCL2* expression was significantly lower in BlCa patients than the controls (Li et al., 2021). Additionally, higher *CCL2* levels were associated with better DFS (Li et al., 2021). In patient tissues, CCL2/*CCL2* was described to be significantly higher in tumors, compared with normal and adjacent tissues (Wang et al., 2017). Considering NMIBC and MIBC patients, higher CCL2 levels significantly correlated with higher grade, stage and lymph node metastasis (Gao et al., 2019). Particularly, considering only MIBC patients, a positive CCL2 expression in tumor cells was associated with poor mean OS, DSS and recurrence-free survival (RFS), while expression of CCL2 in immune cells, was associated with longer OS, DSS, and RFS (Eckstein et al., 2020). The role of CCL2 in immune cells is dependent on the lymph node patient’s status, as CCL2 in N0 was linked to a good prognosis while N1+N2 was associated with poor prognosis (Eckstein et al., 2020) (Table 1).

In urine samples from BlCa patients, advanced stages (pT2-pT4) presented three to fourfold higher mean concentration, comparing with pT1 stage tumors (Amann et al., 1998) (Table 1).

*In vitro*, it was demonstrated that higher CCL2 levels were associated with high-grade BlCa cell lines (T24 and J82), while low-grade BlCa cell lines (SVHUC1, RT4 and TSGH8301), showed lower CCL2 levels (Chiu et al., 2012). In addition, higher CCL2 levels were produced in MB49 and MBT-2 cisplatin-resistant cells lines, comparing with parental BlCa cell lines (Takeyama et al., 2020). So far, there is a lack of information about CCL2 expression in plasma, in *in vivo* and in serum of BlCa patients (Table 1).

Besides, in BlCa, the knowledge about the impact of CCL2 in EMT induction and the signaling pathways activated by CCL2 promoting EMT, is still poor, although some studies have been arising. Co-culture of mast cells (HMC-1) with the BlCa cell lines, T24 and 647V, resulted in an increase of the estrogen receptor beta (ERβ) levels and of CCL2 levels in both cell types (Rao et al., 2016). After co-culture, higher CCL2 levels promoted EMT, driving stimulation of MMP-9 expression and enhanced N-cadherin, Twist, Snail and Vimentin expression levels, resulting in higher BlCa cell lines invasion abilities (Rao et al., 2016) (Table 1). Long noncoding RNA Lymph Node Metastasis Associated Transcript 1 (LNMAT1), overexpressed in BlCa tissues comparing with normal adjacent tissues, can directly interact with heterogeneous nuclear ribonucleoprotein L (hnRNPL), resulting in an increase of the H3 lysine four trimethylation (H3K4me3) of the *CCL2* promoter (Chen et al., 2018). *CCL2* overexpression resulted in increased TAM recruitment. Macrophage activation resulted in secretion of lymphangiogenic growth factor (VEGF-C) to the bladder TME, promoting lymphangiogenic and lymphatic metastasis (Chen et al., 2018). In mouse BlCa cell line MBT2, CCL2-CCR2 interaction may facilitate migration by phosphorylating paxillin y118 through a protein kinase C (PKC)-dependent mechanism (Chiu et al., 2012).

### CXCL1

CXCL1, also known as MGSA, is a powerful neutrophil chemoattractant chemokine (De Filippo et al., 2013; Boro and Balaji, 2017), interacting with the CXCR2 receptor (Kawanishi et al., 2008). CXCL1 plays a double role in immune responses, since it can recruit and activate neutrophils to the infection area, but can also activate the release of several proteases and reactive oxygen species (ROS) that will result in cell death (Sawant et al., 2016). This chemokine plays important roles in several tumor models, promoting cell migration and invasion (Cheng et al., 2011; Wang et al., 2018). Mast cells, alongside with macrophages are able to produce CXCL1 (De Filippo et al., 2013).

*In silico,* UALCAN analysis showed higher *CXCL1* transcript levels in BlCa samples compared with normal bladder mucosa tissues, and, according to GEPIA and GEO database analysis, higher *CXCL1* was significantly associated with shorter OS (Sun et al., 2021b) (Table 1).

While benign or normal bladder tissues showed absent CXCL1 levels, higher CXCL1 levels were significantly associated with more undifferentiated tumors and MIBC (Kawanishi et al., 2008; Miyake et al., 2013). Consequently, high amounts of CXCL1 contributed to poor DSS and poor OS (Miyake et al., 2013). Additionally, increased CXCL1 levels in the tumors promoted the recruitment of CAFs and were associated with higher number of TAMs (Miyake et al., 2016) (Table 1).

In *in vitro* studies, higher *CXCL1* expression was observed in the most aggressive BlCa cell lines (UMUC3, 5637 and T24) (Kawanishi et al., 2008). Moreover, CXCL1 could enhance the invasive ability of BlCa cell lines (Kawanishi et al., 2008; Miyake et al., 2019). Additionally, CXCL1 influenced the angiogenesis process and tumor vasculature, since tube structures were significantly lower after treatment with conditioned media from *CXCL1*-knockdown T24 cells (Miyake et al., 2019). Furthermore, higher CXCL1 amounts were obtained with MB49, MBT-2 and T24 cisplatin-resistant cells lines, in comparison with parental BlCa cell lines (Takeyama et al., 2020) (Table 1).

*In vivo*, it was shown that CXCL1 secreted by TAMs and CAFs enhanced bladder tumor cell attachment to the bladder wall, consequently inducing tumor growth (Miyake et al., 2016). Moreover, by using T24 cell xenografts treated with HL2401, a CXCL1 inhibitor, it was observed a significant increase in the apoptotic index, but a significant decrease in microvessel density and a reduction in proliferation (Miyake et al., 2019).

In liquid biopsies, CXCL1 urinary protein concentrations were significantly higher in BlCa patients comparing with patients without BlCa (Kawanishi et al., 2008; Burnier et al., 2015). Importantly, a significant increase was obtained in stages pT1-pT4, comparing with pTa (Kawanishi et al., 2008) (Table 1).

Information regarding CXCL1 expression in serum and in plasma of BlCa patients is still lacking (Table 1). Also, the role that CXCL1 might have in driving EMT is little explored, as well as the signaling pathways activated by CXCL1 to induce EMT in BlCa. However, it is known, that *in vivo*, overexpression of CXCL1 by TAMs and CAFs, promoted alterations in BLCa EMT, decreasing E-cadherin membrane expression, while increasing MMP-2 expression (Miyake et al., 2016) (Table 1). Furthermore, in tissues, a significant correlation was established between CXCL1 and MMP-13 (Kawanishi et al., 2008) (Table 1). *In silico* analysis, using LinkedOmics database, also showed that microRNA (miR)-200a, an important hallmark in EMT (Adam et al., 2009), interacts with *CXCL1* (Sun et al., 2021b) (Table 1).

### CXCL12

CXCL12, also known as stromal cell-derived factor 1 (SDF-1), or pre-B cell stimulating factor (PBSF) (Yang et al., 2015), interacts with CXCR4 and CXCR7 receptors (Shen et al., 2013; Zhang et al., 2018). CAFs are able to secrete CXCL12, being essential for CD8^+^ T cells recruitment (Du et al., 2021c). This chemokine participates in the homeostatic regulation of leukocyte trafficking and tissue regeneration (Barinov et al., 2017). CXCL12 is also described to be involved in tumor growth, angiogenesis and tumor cell intravasation (Chang et al., 2020).

*in silico* analyses (GEO, TCGA, ONCOMINE and UALCAN) showed that *CXCL12* was significantly decreased in BlCa samples, comparing with the controls (Sun et al., 2021b; Du et al., 2021c). On the other hand, higher *CXCL12 e*xpression was significantly associated with more advanced stages, worse prognosis, and more lymph node metastasis (N2 showed higher *CXCL12* than N0) (Sun et al., 2021b; Liu et al., 2021).

In accordance with *in silico* data, Du Y *et al.* showed a CXCL12 reduction in BlCa patient tissues comparing with the normal tissues Du et al. (2021c), while Yang DL *et al.* showed a significantly higher expression of CXCR4/CXCL12 in BlCa tissues and no expression in normal tissues Yang et al. (2015). It was demonstrated that CXCL12 positively associated with tumor grade and stage in BlCa patient tissues, being CXCL12 expression more intense in recurrent patients (Batsi et al., 2014). Moreover, Yang DL *et al.* showed that CXCR4/CXCL12 levels strongly associated with tumor progression and invasion, and *CXCL12* transcript levels in tumor tissues increased with tumor aggressiveness.

There are several *CXCL12* mRNA variants depending on alternative splicing (Gosalbez et al., 2014; Chang et al., 2020). CXCL12-α, CXCL12-β and CXCL12-γ are some of the variants, presenting the same first three exons (Chang et al., 2020). According to the literature, CXCL12-α has the strongest affinity to CXCR4, followed by CXCL12-β and CXCL12-γ (Chang et al., 2020). By qPCR, it was demonstrated that CXCL12-α and CXCL12-β levels were higher in metastatic patient tissues compared to non-metastatic patient tissues (Gosalbez et al., 2014). Moreover, only CXCL12-β was significantly higher in tumor patients than normal samples (Gosalbez et al., 2014). In urine, CXCL12-γ was not detected, but CXCL12-α levels were significantly lower in patients with low-grade compared to controls, while CXCL12-β levels were significantly higher in high-grade than the controls (Gosalbez et al., 2014).

There is no information regarding CXCL12 expression in plasma and in serum, similarly to CCL2 and CXCL1 (Table 1). Up till now, CXCL12 has been described to have an important role in regulating some EMT-related molecules in BlCa. Additionally, studies on the signaling pathways that might be activated by this chemokine started to arise. *In vitro,* it was shown that CXCL12 was involved in cell invasion and migration (Retz et al., 2005; Shen et al., 2013). CXCR4 and CXCL12 binding drives the induction of STAT3 phosphorylation (Shen et al., 2013), an important molecule in promoting BlCa growth and survival, and able to work as a transcription factor regulating EMT (Chen et al., 2008; Jin, 2020) (Table 1). This alteration in migration might occur due to an association of CXCR4/CXCL12 with cytoskeletal reorganization, specifically, with a redistribution of F-actin stress fibers (Retz et al., 2005). A study from Zhang T *et al.* reinforced these findings, since SW780 treated with AMD34635, a CXCR4 inhibitor, exhibited growth and colony formation supression, as well as, inhibiton on migration and invasion (Zhang et al., 2018). In addition, *in vivo*, it was demonstrated that tumors with AMD3465-treatment showed slower growth and lower weight than tumors treated with the vehicle (Zhang et al., 2018). Additionally, *in vitro*, it was also demonstrated that molecular alterations occurred, with a decrease of β-catenin, MMP-2 and c-Myc expression and with an increase in E-cadherin levels (Zhang et al., 2018) (Table 1). However, the effect of AMD3465 was reversed when CXCL12 was added, inducing E-cadherin downregulation and c-Myc upregulation (Zhang et al., 2018) (Table 1). Moreover, SW780 cells treated with FH535, a β-catenin antagonist, also decrease cell proliferation, colony formation, migration and invasion, being these effects once again reverse by CXCL12 treatment. Thus, suggesting that CXCR4/CXCL12 play an important role in regulated β-catenin expression in BlCa progression (Zhang et al., 2018) (Table 1).

### IL-6

IL-6 is a pro-inflammatory interleukin (Chen et al., 2013; Morizawa et al., 2018) known to play a major role in inflammatory responses (Chen et al., 2013; Yao et al., 2020), as well as in the maturation of B cells (Andrews et al., 2002; Miyake et al., 2019). IL-6 binds to the receptor IL6-R, present in the extracellular membrane, or secreted in a soluble form (IL-6sR) (Andrews et al., 2002). IL-6 is mainly produced by tumor-infiltrating immune cells, such as T cells and macrophages, by tumor cells, by healthy endothelial tissues, by epithelial cells and by muscle cells (Andrews et al., 2002; Rossi et al., 2015; Schuettfort et al., 2022).

In tissues, Chen MF *et al.* showed that IL-6/*IL6* expression was higher in BlCa tissues, comparing with non-malignant tissues (Chen et al., 2013). The authors demonstrated that non-malignant tissues exhibited IL-6 expression, but in lower levels, compared to early stages, while IL-6 higher levels were mostly associated with MIBC tissues (Chen et al., 2013) (Table 1).

*In silico* analysis, revealed that *IL6* transcript levels were significantly increased in higher stages (stages III and IV), comparing with lower stages (stages I and II) (Goulet et al., 2019). Moreover, *IL6* was significantly enhanced in high-grade patients, comparing with low-grade patients (Goulet et al., 2019) (Table 1).

In urine samples, IL-6 levels were significantly higher in advanced stage patients (pT3-pT4), comparing with patients with early stage tumors or non-malignant samples (Chen et al., 2013). Kumari N *et al.* showed that higher IL-6 concentration was significantly associated with lower disease grade Kumari et al. (2017). Furthermore, it was demonstrated that IL-6 levels in urine were associated with shorter OS (Morizawa et al., 2018) (Table 1).

Using preoperative plasma samples, Schuettfort VM *et al.* and Andrews B *et al.* demonstrated that IL-6 and IL-6sR were significantly higher in patients with advanced stages, lymph vascular invasion and lymph node metastasis Andrews et al. (2002), Schuettfort et al. (2022). Dmytryk V *et al.* also observed significantly higher IL-6 leveles in pT3-pT4 samples, comparing with control samples Dmytryk et al. (2020). Moreover, patients with recurrent disease or patients who deceased due to BlCa disease presented higher IL-6 and IL-6sR levels (Schuettfort et al., 2022). Higher IL-6 and IL-6sR levels were associated with poor RFS, CSS and OS (Schuettfort et al., 2022). Andrews B *et al.* showed that plasma IL-6 levels were significantly higher in BlCa than in healthy patients, however IL-6sR levels did not present statitiscal differences bteween the two groups (Andrews et al., 2002) (Table 1).

In serum BlCa samples, collected prior to surgery, IL-6 levels were significantly higher in recurrent patients, comparing with non-recurrent patients and were significantly associated with poor RFS (Kumari et al., 2017). Similar to IL-8, IL-6 expression was significantly associated with shorter CSS (Morizawa et al., 2018). However, Yang G *et al*. described a descrease of IL-6 levels in T2-T4 patient samples, comparing with Ta-T1 samples and healthly controls (Yang et al., 2017) (Table 1).

Regarding the literature, BlCa cell lines produced high IL-6 levels, while normal cell lines expressed only low IL-6 levels (Okamoto et al., 1997). Upon IL-6 treatment, BlCa cell lines (253J, RT4 and T24) presented enhanced cellular growth, comparing with normal cell lines (Okamoto et al., 1997). Moreover, the cell growth was significantly inhibited upon anti-IL-6 neutralizing antibody treatment, suggesting that IL-6 provides autocrine growth advantages to the BlCa cell lines (Okamoto et al., 1997). Additionally, Yeh CR *et al.* suggested that, *in vitro*, ERα overexpression in fibroblasts may increase BlCa cell invasion through IL-6 expression in BlCa cells (Yeh et al., 2015). Miyake M *et al.* demonstrated, *in vivo* and *in vitro,* that CXCL1 had an important impact in BlCa tumor growth, since promoted IL-6 induction and repressed tissue inhibitor of metalloproteinase 4 (TIMP4) inhibition Miyake et al. (2019). Chen MF *et al.* showed that *IL6* silencing contributed to a decrease in tumor invasion and tumor growth/proliferation, both *in vivo* and *in vitro* (HT1197 and HT1376 cell lines) Chen et al. (2013).

Overall, IL-6 has been described to be upregulated in advanced BlCa patients (Chen et al., 2013; Goulet et al., 2019) and in lymph node metastasis (Andrews et al., 2002; Schuettfort et al., 2022). Thus, the association between IL-6 and EMT induction starts to be studied in BlCa, along with which signaling pathways can be activated by IL-6. Indeed, *IL6* overexpression in HT1376 cells promoted a decrease in N-cadherin and Vimentin levels, while the *IL6* knockdown in T24 cells led to a decrease in E-cadherin, but an increase in N-cadherin and Vimentin levels (Tsui et al., 2013) (Table 1). However, it was demonstrated that *IL6* silencing was able to increase E-cadherin levels, but decreased MMP-9 levels and attenuated angiogenesis, since it led to a decrease of CD31 and vascular endothelial growth factor (VEGF) levels (Chen et al., 2013) (Table 1). EMT-player alterations, induced by IL-6, might be regulated by STAT3 signaling pathway activation (Chen et al., 2013) (Table 1). In patient tissues, it was demonstrated a significant positive correlation between p-STAT3 Y705 and IL-6, and a significant negative correlation between p-STAT3 Y705 and E-cadherin, suggesting that E-cadherin expression might be inhibited by IL6-STAT3 signaling pathway (Chen et al., 2020). *In vitro*, it was demonstrated that IL-6-induced STAT3 is able to target *TWIST* promoter, modulating EMT and BlCa cell invasion (Yao et al., 2020).

### TGF-β1

TGF-β1 is the most well studied isoform and its receptors are membrane serine-threonine kinase receptors I and II (TGF-βRI and TGF-βRII) (Kim et al., 2001). This cytokine has been described as playing a dual role in tumorigenesis, displaying a tumor suppressor role in normal cells or in early tumor stages, inducing cell cycle arrest and apoptosis, while in late stages can promote cell motility and invasion (Eder et al., 1997; Jakowlew, 2006; Lebrun, 2012; Stojnev et al., 2019). Overall, TGF-β1 is mainly released by regulatory T cells (Efiloğlu et al., 2020) and CAFs (Ao et al., 2007; Yu et al., 2014; Yeh et al., 2015), M2 macrophages (Horibe et al., 2021) and MDSC (Groth et al., 2019). TGF-β1 can activate both SMAD-dependent or SMAD-independent signaling (Hata and Chen, 2016). TGFRβII point mutations have been reported, not only in the BlCa cell line T24, but also in BlCa patients, being associated with higher pathologic T category and tumor grade (Bian et al., 2013).

*In silico* analysis, it was demonstrated that *TGFB1* is upregulated in MIBC compared to NMIBC and patients with higher *TGFB1* expression presented higher risk of death, lower DFS and lower OS (Zou et al., 2019) (Table 1).

In BlCa patient samples, TGF-β1 is expressed in normal urothelium, although at lower levels (Yang et al., 2018; Zou et al., 2019). Within tumors, higher TGF-β1 levels were significantly associated with higher tumor stage and grade and correlated with cancer-specific death (Kim et al., 2001; Yang et al., 2018; Stojnev et al., 2019; Zou et al., 2019). On the other hand, although Miyamoto H *et al.* also found that *TGFB1* transcript levels were higher in tumor tissues, than in normal samples, *TGFB1* transcript levels were significantly associated with low-grade and stage Miyamoto et al. (1995) (Table 1).

In BlCa patient serum samples, Efiloğlu Ö *et al.* described that higher TGF-β1 was associated with a low risk of tumor progression (Efiloğlu et al., 2020). Indeed, Eder IE *et al.*, using serum samples, mentioned that TGF-β1 levels were significantly lower in T4 tumors than superficial and invasive (T2-T3) tumors Eder et al. (1997). However, Eder IE *et al.* demonstrated that superficial tumors (Ta-T1) had significantly TGF-β1 higher levels, than normal samples (Eder et al., 1997). Another study from Eder IE *et al.* mentioned that serum TGF-β1 were elevated in the most aggressive BlCa cases compared to controls, and in the most undifferentiated tumors, than with lower grade tumors (Eder et al., 1996) (Table 1).

Also in preoperative plasma, TGF-β1 levels were significantly higher in MIBC patients with regional and distant lymph node, comparing with non-metastatic MIBC and controls (Shariat et al., 2001). An increase of TGF-β1 was found in MIBC, comparing with less aggressive tumors, with patients with higher TGF-β1 demonstrating increased risk of disease recurrence and mortality (Shariat et al., 2001). On the other hand, no significant differences were found between controls and patients with early stages (Shariat et al., 2001) (Table 1).

In urine samples, it was observed a significantly higher number of BlCa samples expressing TGF-β1 comparing with chronic cystitis disease cases or the control group (Helmy et al., 2007) (Table 1).

*In vivo*, it was observed an increase of, not only in tumor size, but also in tumor weight (Zou et al., 2019) when the 5637 cell line overexpressing TGF-β1 was transplanted into mice, compared with the parental cell line (Zou et al., 2019) (Table 1).

As mentioned above, TGF-β1 is an important inducer and regulator of EMT (Stojnev et al., 2019). EMT-related molecules regulated by TGF-β1 and the signaling pathways activated by this cytokine have been well described in several models, including in BlCa. Both *in vitro and in vivo*, an increase of TGF-β1 reflected in an upregulation of EMT-related molecule levels, such as Slug, Vimentin, Snail, MMP-2, MMP-9 and E-cadherin (Zou et al., 2019). Additionally, TGF-β1 has been associated with proliferation, colony formation, migration and invasion in BlCa cell lines (Bian et al., 2013; Zhang et al., 2016; Zou et al., 2019). HTB9 and T24 cell lines treated with TGF-β1 resulted in E-cadherin/*CDH1* decrease, and a N-cadherin/*CDH2* and Vimentin/*VIM* increase (Chen et al., 2014). Upon TGF-β1 treatment, it was shown *miR-200b* downregulation and MMP-16 upregulation, due to miR-200b targeting of MMP-16 (Chen et al., 2014). TGF-β1 treatment of T24 and BIU87 BlCa cell lines resulted in increased fascin1 levels, an important molecule in tumor migration and invasion (Zhang et al., 2016). Finally, AY-27, a rat cell line, treated with TGF-β1 resulted in alterations in morphology, with the increase of spindle shaped cells, while the polygonal shaped cells decreased, as well as cell-to-cell contact (Koo et al., 2010). In Smad-dependent signaling, it occurs recruitment and phosphorylation of SMAD2 and SMAD3 (Heldin et al., 2012; Gonzalez and Medici, 2014; Papageorgis, 2015; Gupta et al., 2016). Then, SMAD4 is recruited, forming a trimeric complex capable to be translocated to the nucleus (Bian et al., 2013; Gonzalez and Medici, 2014; Gupta et al., 2016). In BlCa samples, Smad2 and Smad4 expression were associated with low-grade and superficial tumors, and better overall survival of the patients (Stojnev et al., 2019). However, it was observed an increase of p-SMAD2 in invasive bladder tumors (Gupta et al., 2016). Knockdown of *PPM1A*, an antagonist of TGF-β signaling by dephosphorylating TGF-β-activated Smad2/3, resulted in an increase in p-Smad2/3 levels upon TGF-β1 treatment, in 5637 and T24 cell lines (Geng et al., 2014).

## Conclusion

**FIGURE 1:**
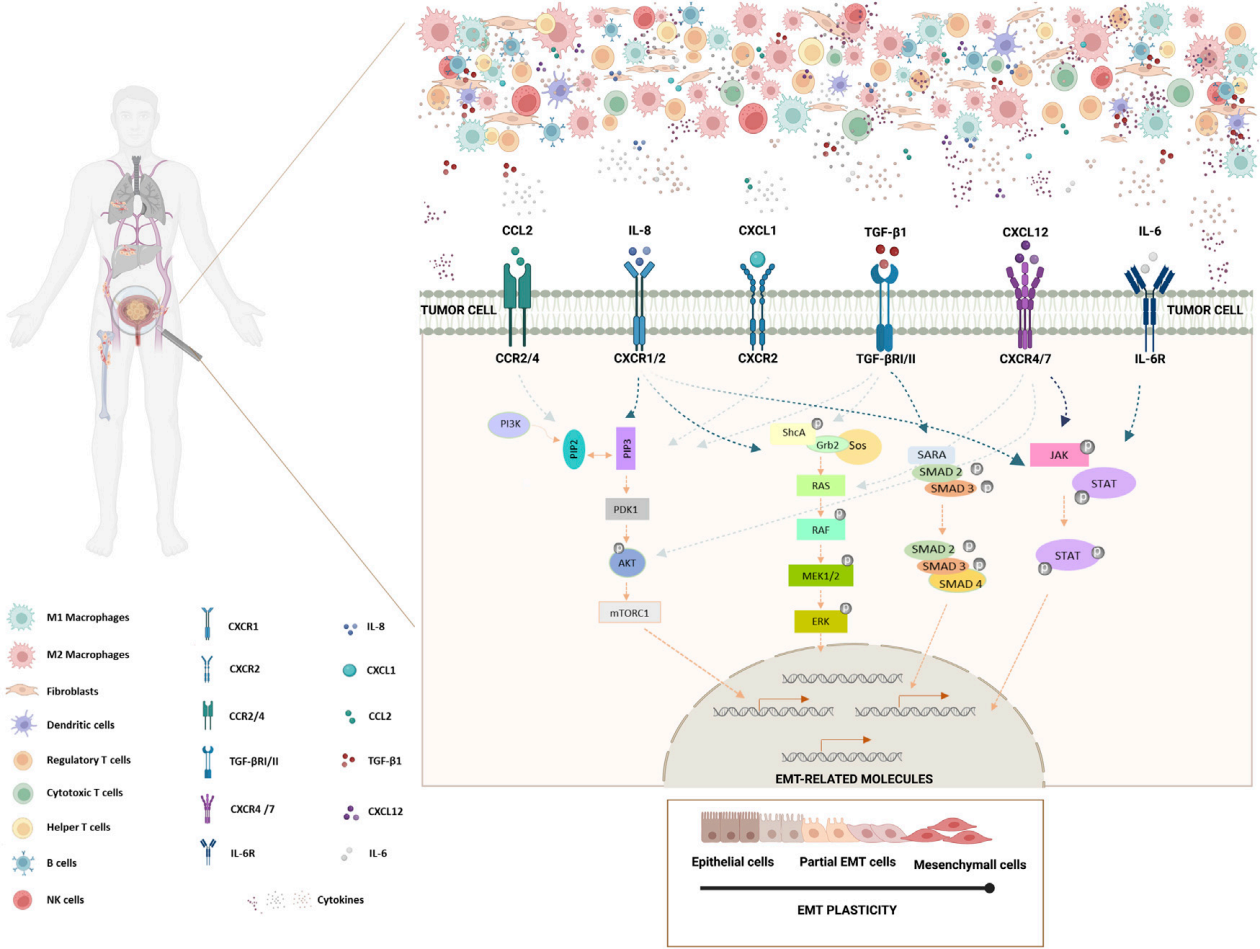
Schematic representation of the impact of BlCa TME cytokines/chemokines in EMT induction in bladder tumor cells. Bladder tumor microenvironment is comprised by tumor cells and several tumor-infiltrating immune cells, such as, M1 and M2 macrophages, dendritic cells, regulatory T cells, cytotoxic T cells, helper T cells, B cells and NK cells. Furthermore, TME includes stromal cells, like fibroblasts, and non-cellular components, including soluble biological factors or mediators, as cytokines/chemokines. Cytokines/chemokines are mainly produced by several immune cells and fibroblasts, but they also can be produced by tumor cells. Tumor cells present several cytokine/chemokine receptors. IL-8 binds to CXCR1/CXCR2 receptors, CCL2 binds to CCR2/CCR4 receptor, TGF-β1 binds to TGF-βRI/II receptors, CXCL1 binds to CXCR2 receptor, CXCL12 binds to CXCR4/7 receptors and IL-6 binds to IL-6R receptor. Cytokine/receptor binding on tumor cells can drive the deregulation of specific molecules, including the triggering of EMT signaling pathways. Here, are depicted the most relevant signaling pathways involved in driving EMT that have been described to be deregulated in BlCa upon cytokine binding. JAK-STAT, RAS-RAF-ERK and AKT signaling pathways and TGF-β SMAD-dependent pathway are described to play roles in the activation of EMT-related molecules, driving EMT processes in tumor cells. Bladder tumor cells presenting partial EMT demonstrate a higher survival mechanism and a higher tumor-initiating and metastatic potential. In this way, bladder tumor cells are able to metastasize to the bones, lungs and liver (Created with BioRender.com).

In this review, we focused on the dysregulation of several immune cells, and of key cytokines/chemokines in the bladder cancer TME. In BlCa, IL-6, CCL2, CXCL1, CXCL12, IL-8 and TGF-β1 play putative roles in promoting tumor progression, growth, invasion, and metastases formation (Figure 1). The cytokine-driven modulation of the transcription of specific EMT-related molecules in BlCa starts to be unravel (Figure 1). However, the mechanisms involved in the axis TME-EMT signaling pathway activation in BlCa remains to be further exploited. Therefore, finding novel cytokines/chemokines present in bladder TME driving EMT induction and, simultaneously, decipher crucial players involved in BlCa tumorigenesis and progression.
